# Traditional eye medicine use in microbial keratitis in Uganda: a mixed methods study

**DOI:** 10.12688/wellcomeopenres.15259.2

**Published:** 2019-10-04

**Authors:** Simon Arunga, Allen Asiimwe, Eunice Apio Olet, Grace Kagoro-Rugunda, Bosco Ayebazibwe, John Onyango, Robert Newton, Astrid Leck, David Macleod, Victor H. Hu, Janet Seeley, Matthew J. Burton

**Affiliations:** 1 Department of Ophthalmology, Mbarara University of Science and Technology, Mbarara, Uganda; 2 International Centre for Eye Health, London School of Hygiene & Tropical Medicine, London, UK, WCIE 7TH, UK; 3 MRC/UVRI, LSHTM Uganda Research Unit, Entebbe, Entebbe, Uganda; 4 Department of Biology, Mbarara University of Science and Technology, Mbarara, Uganda; 5 Ruharo Eye Centre, Ruharo Mission Hospital, Mbarara, Uganda; 6 University of York, UK, York, YO10 5DD, UK; 7 Tropical Epidemiology Group, London School of Hygiene & Tropical Medicine, London, UK, WCIE 7TH, UK; 8 Department of Global Health and Development, London School of Hygiene & Tropical Medicine, London, UK, WCIE 7TH, UK

**Keywords:** Microbial Keratitis, Traditional Eye Medicine, Traditional Healers, Blindness, Uganda

## Abstract

**Background**: Traditional eye medicine (TEM) is frequently used to treat microbial keratitis (MK) in many parts of Africa. Few reports have suggested that this is associated with a worse outcome. We undertook this large prospective study to determine how TEM use impacts presentation and outcome of MK and to explore reasons why people use TEM for treatment in Uganda.

**Methods**: In a mixed method prospective cohort study, we enrolled patients presenting with MK at the two main eye units in Southern Uganda between December 2016 and March 2018 and collected information on history, TEM use, microbiology and 3-month outcomes. We conducted qualitative interviews with patients, carers traditional healers on reasons why people use TEM. Outcome measures included presenting vision and at 3-months, comparing TEM Users versus Non-Users. A thematic coding framework was deployed to explore reasons for use of TEM.

**Results**: Out of 313 participants enrolled, 188 reported TEM use. TEM Users had a delayed presentation; median presenting time 18 days versus 14 days, p= 0.005; had larger ulcers 5.6 mm versus 4.3 mm p=0.0005; a worse presenting visual acuity median logarithm of the minimum angle of resolution (Log MAR) 1.5 versus 0.6, p=0.005; and, a worse visual acuity at 3 months median Log MAR 0.6 versus 0.2, p=0.010. In a multivariable logistic regression model, distance from the eye hospital  and delayed presentation were associated with TEM use. Reasons for TEM use included lack of confidence in conventional medicine, health system breakdown, poverty, fear of the eye hospital, cultural belief in TEM, influence from traditional healers, personal circumstances and ignorance.

**Conclusion**: TEM users had poorer clinical presentation and outcomes. Capacity building of the primary health centres to improve access to eye care and community behavioural change initiatives against TEM use should be encouraged.

## Introduction

Microbial keratitis (MK) frequently leads to sight-loss from dense corneal scarring, or even loss of the eye, especially when the infection is severe and/or appropriate treatment is delayed ^1^. MK has been described as a “silent epidemic”, which leads to substantial morbidity, related to blindness and other consequences such as pain and stigma ^2^. It is the leading cause of unilateral blindness after cataract in tropical regions and is responsible for about 2 million cases of monocular blindness per year ^3^.

In Low and Middle-Income Countries (LMIC), use of Traditional Eye Medicine (TEM) for treatment of many eye conditions is a common practise ^4– 6^. In the few reported studies, TEM has been found to lead to complications such as corneal scarring and delayed presentation of patients to hospital resulting in poor outcomes ^7, 8^.

Literature on TEM use for MK is scanty. However, among the three papers from Sub-Saharan Africa (SSA), TEM use among patients with MK was reported to be associated with a severe presentation. These studies did not report clinical outcomes ^9– 11^. In addition, since most of the TEM involves plant products such as fresh leaves, it could have a major role in the pathogenesis of fungal keratitis, which has been associated with injuries involving vegetative matter ^12, 13^. Our experience in Uganda is that TEM is widely used to treat a number of eye conditions including MK. However, the drivers of this practice are not well understood.

The aim of this study therefore was to determine how TEM use impacts presentation and outcome of MK and to explore reasons why people use TEM for treatment of MK in Uganda.

## Methods

### Ethical statement

This study adhered to the Declaration of Helsinki. It was approved by the London School of Hygiene & Tropical Medicine Ethics Committee (Ref 10647), Mbarara University Research Ethics Committee (Ref 10/04-16) and Uganda National Council for Science and Technology (Ref HS-2303). Written informed consent in Runyankore, the local language, was obtained before enrolment. If the patient was unable to read, the information was read to them, and they were asked to indicate their consent by application of their thumbprint. The collected source data is stored in a secure database at Mbarara University of Science and Technology. An anonymised digital version was also uploaded in a secure server. The data will be kept for 7 years according to institutional policy.

### Participants

Due to the cultural complexity of TEM usage, we used a mixed methods approach. We prospectively enrolled patients with MK that consecutively presented to two tertiary eye hospitals in South-Western Uganda from December 2016 to March 2018. The case definition of MK was the presence of a corneal epithelial defect (of at least 1mm diameter) with an underlying stromal infiltrate, associated with signs of inflammation (conjunctival hyperaemia, anterior chamber inflammatory cells, +/- hypopyon). We excluded those not willing to participate, those not willing to return for follow-up, pregnant women, lactating mothers, those aged below 18 years.

### Quantitative assessment

We documented basic demographic information and ophthalmic history using ophthalmic nurses as part of the routine hospital work up. This included treatment received including prior use of TEM. For those who reported use of TEM, a detailed structured history was taken on what they had applied, source of the medicines, cost, how it was prepared, duration of use and any complications experienced. A detailed description of the cases evaluation has been previously presented. In summary, after measurement of the presenting visual acuity (Logarithm of Minimum Angle of Resolution), cases underwent a detailed clinical examination on a slit lamp using a structured protocol, including eyelid assessment, corneal ulcer features, anterior chamber (flare, cells, hypopyon shape and size) and perforation status. Corneal scrapes were collected for microscopy, culture (blood agar, chocolate agar, potato dextrose agar) and molecular diagnosis. HIV, Diabetes counselling and testing were offered, as per the Uganda Ministry of Health HIV testing protocol. Cases were treated according to the hospital protocol, which usually involved a brief admission for the first few days. The study follow-up assessment schedule was days 2, 7, 21 and 90, to determine outcome. Patients were asked to return to the eye hospital for these reviews where their follow up data was collected as before. Additional assessments were conducted as clinically indicated. The primary outcome measure was final best corrected vision at 3 months. See extended data ^14^ for questionnaire used.

### Qualitative assessment

All interviews and discussion groups were conducted by AA. They were audio recorded and summarised. Additional contextual information provided such as patient emotions, environment and any other aspect the interviewer found noteworthy.

Firstly, at presentation, patients who reported to have used TEM were asked if they would be willing to discuss their experiences. For such patients, an interviewer would return later that evening or the next day when the patient was more relaxed. Interviews were conducted in the local language by a social scientist either at the hospital bedside (when quiet) or in the hospital compound depending on the patient’s preference. The focus of the interview was to explore reasons why they had used TEM.

Secondly, we conducted informal group discussions (IGDs) with a sample of the MK patients involved in the study and relatives of people with MK on the practise and reasons why people use TEM. This was an opportunistic approach to allow flexible data collection. For example, a patient might present escorted by many family members and friends (common in this setting), such a group would then be invited to discuss issues around TEM. Such a naturally composed group was to result in a more relaxed discussion than a group of people who did not know each other who are brought together solely for the discussion.

Finally, we conducted in-depth interviews with traditional healers to learn about what they would usually do for people presenting with a problem like MK and why people go to them for treatment. Healers were identified from a traditional healers’ registry at the local council headquarters. A random sample of 15 traditional healers were contacted through their coordinator. Those willing to share their knowledge and practise in treating eye problems particularly MK were visited and interviewed at their home or shrine.

For all the groups, topic guides were developed using available literature and experiences of the local ophthalmologists treating patients with MK (see extended data ^14^). They included local understanding of MK, causes, treatment and experiences of using TEM. The guides were piloted among a few patients and modified accordingly. The final version was approved by all the authors who included senior social scientists (AA) and a professor (JS). In this report, our focus is on reasons why people use/do not use TEM. These were reviewed by one of the authors. They were then piloted among MK patients and revised accordingly. All interviews lasted about 30–45 minutes.

### Analysis

Quantitative data were analysed using STATA v14. We compared demographic data, baseline clinical presentation and final vision outcomes at 3-months of patients who reported to have used TEM versus those who had not. Appropriate tests of significance (chi2 for categorical data and Wilcoxon rank sum for continuous data) were employed. Multivariable logistic regression analysis was used to identify factors associated with TEM use. Initially, univariable regression was performed to generate crude odds ratios (OR). Variables with a p-value less than 0.1 were introduced in the multivariable model. A back stepwise approach was then used, until only the variables with a p-value of less than 0.05 were retained. Adjusted OR were reported for the final model. Summary tables of proportions were constructed to describe the source, cost, complications and duration of use of TM.

For the qualitative data, all interviews were recorded with an audio recorder (Olympus WS-853 Digital Stereo Voice Recorder) and transcribed into summaries. These were independently reviewed several times by two of the authors (SA and JS). A coding framework was developed, and data were then manually coded. Emerging themes around reasons why people used/did not use TEM are presented. Specific conversation response clips from the respondents that supported the generated themes were extracted from the audio recordings and used as illustrative statements.

## Results

We enrolled 313 people with MK, of whom 188 (60%) reported TEM use (“TEM Users”) and 125 said they did not use TEM (“TEM Non-Users”). The demographic characteristics of both groups are shown in Table 1 (see underlying data ^14^). There were some differences between TEM Users and Non-Users. TEM Users lived further from the eye unit, were more frequently farmers, were less likely to be married and had progressed less in formal education.

**Table 1.** Baseline demographics characteristics of participants (n=313), comparing traditional eye medicine (TEM) users to non-users.

Variable		TEM Users (188)			TEM Non-Users (125)			
		Median	(IQR)	(Total range)	Median	(IQR)	(Total range)	P value
**Age**		48	(34–60)	(18–87)	45	(35–60)	(18–96)	0.651
**Distance to eye hospital (km)**		87	(59–132)	(1.5–378)	67	(42–121)	(0.2–316)	0.003
**Distance to nearest Health Centre in (km)**		3	(1–5)	(0–45)	2	(1–4)	(0–35)	0.528
		Count	(%)		count	(%)		P value
**Gender**	Male	101	(54)		73	(58)		0.415
**Occupation**	Farmer	140	(75)		80	(64)		0.047
	Non-farmer	48	(25)		45	(34)		
**Education**	None	59	(31)		25	(20)		0.016
	Primary Level	98	(52)		64	(51)		
	Secondary Level	23	(12)		22	(18)		
	Tertiary Level	8	(5)		14	(11)		
**Marital status**	Unmarried *	66	(35)		29	(23)		0.025
	Married	122	(65)		96	(77)		
**Household SES Ɨ**	Poor	51	(28)		34	(29)		0.520
	Middle	116	(64)		72	(60)		
	Upper	13	(7)		13	(11)		

SES: Socioeconomic status. *Unmarried included-single, divorced, widowed. Ɨ This was relative self-reported economic status compared to the neighbours.

The clinical characteristics of both groups are shown in Table 2. There was evidence that the condition of TEM Users was worse than TEM Non-Users at presentation. The TEM Users presented later, had larger corneal ulcers (both infiltrate and epithelial defect), more frequent hypopyons and poorer vision.

**Table 2.** Baseline clinical characteristics of participants (n=313), comparing traditional eye medicine (TEM) users to non-users.

Variable		TEM Users (188)			TEM Non-Users (125)			
		Median	(IQR)	(Total range)	Median	(IQR)	(Total range)	P value
**Presentation time in days**		18	(12–35)	(1–274)	14	(5–32)	(0–370)	0.005
Infiltrate size in mm *		5.6	(3.8–8.1)	(0.5–11)	4.3	(2.4–6.8)	(0.6–12)	0.0005
Epithelial defect size in mm *		4.2	(2.5–11)	(0–14)	3.6	(2.2–5.1)	(0–11)	0.0105
**Presenting Vision (Log MAR)**		1.5	(0.3–2.5)	(0–4)	0.6	(0.2–2.5)	(0–4)	0.005
		Count	(%)		count	(%)		P value
**Visual Acuity**	> 6/18	50	(27)		52	(42)		0.011
	6/18 – 6/60	24	(13)		18	(14)		
	< 6/60	113	(60)		55	(44)		
**Eye discharge**	Yes	107	(57)		60	(48)		0.122
**History of Trauma**	Yes	42	(22)		49	(39)		0.001
**Presence of lid swelling**	Yes	85	(46)		45	(36)		0.097
**Slough Ɨ**	None	31	(17)		30	(24)		0.246
	Flat	77	(41)		47	(38)		
	Raised	78	(42)		46	(37)		
**Infiltrate colour**	White	77	(44)		71	(63)		0.005
	Cream	76	(43)		30	(27)		
	Other	23	(13)		11	(10)		
**Hypopyon**	Yes	66	(35)		28	(22)		0.014
**Perforated at admission**	Yes	29	(15)		16	(13)		0.517
**Microbiology**	Unknown	38	(23)		27	(25)		0.089
	Bacteria	10	(6)		10	(10)		
	Fungus	108	(67)		60	(55)		
	Mixed	6	(4)		11	(10)		

Log MAR: Logarithm of the minimum angle of resolution.

*These were calculated as the geometrical means using the MUTT protocol ^15^. The upper limits exceeded normal corneal diameter for some lesions, which extended up to the sclera. Ɨ Raised slough was when the corneal infiltrate profile was raised, flat slough was when the profile was flat while no slough is when there was no debris noted. The difference in presenting vision and infiltrate sizes remained significant even after adjusting for delayed presentation.

We modelled factors associated with TEM use ( Table 3). After adjusting for potential confounders, distance from the eye hospital and delayed presentation were associated with TEM use. Whereas, there was less TEM use among those who were married, had a history of trauma and a high education level.

**Table 3.** Univariable and multivariable logistic regression for factors associated with traditional eye medicine use (n=313).

Variable	Univariable Analysis			Multivariable Analysis		
	Crude OR	(95% CI)	p-value	Adjusted OR	(95% CI)	p-value
**Age in years**	1.002	(0.988-1.016)	0.699			
**Distance to Eye hospital (for every km)**	1.005	(1.001-1.0090	0.009	1.004	(1.001-1.008)	0.035
**Distance to the nearest Health Centre (for every km)**	1.028	(0.971-1.089)	0.332			
**Sex (Being male)**	0.82	(0.52-1.30)	0.415			
**Occupation (Being a farmer)**	1.64	(1.01-2.68)	0.048			
**Married**	0.55	(0.33-0.93)	0.026	0.54	(0.31-0.95)	0.035
**Education level**						
None	1		0.016	1		0.059
Primary	0.64	(0.36-1.14)		0.71	(0.38-1.30)	
Secondary	0.44	(0.20-0.93)		0.44	(0.20-1.00)	
Tertiary	0.24	(0.09-0.65)		0.28	(0.09-0.83)	
**Household economic status**						
Low	1		0.526			
Middle	1.07	(0.63-1.81)				
Upper	0.66	(0.27-1.61)				
**Presentation time**						
0–3 days	1		<0.001	1		0.002
4–7 days	2.17	(0.72-6.53)		1.50	(0.46-4.83)	
8–14 days	6.03	(2.10-17.3)		4.76	(1.55-14.6)	
15–30 days	5.77	(2.03-16.4)		4.37	(1.44-13.2)	
>30 days	4.89	(1.75-13.6)		3.74	(1.27-11.1)	
**History of trauma**	0.44	(0.26-0.72)	0.001	0.43	(0.25-0.74)	0.003

At 3-months, 260 patients completed their follow-up. There was no systematic baseline difference between patients who were seen at 3-months and those that were not. The final LogMAR visual acuity was worse among TEM Users, median 0.6 (IQR 0-2.5), compared to TEM Non-Users, 0.2 (IQR 0-1.5), p=0.010.

Among the 188 patients who reported TEM use, 137 (73%) used TEM after they had been to a government health facility (secondary TEM use). TEM was mostly made from fresh leaves [154, (82%)]; the commonest preparation method was to freshly squeeze them [145, (77%)]. Most patients obtained TEM either from their home garden (40%) or from a neighbour (54%), only 5 patients (3%) obtained TEM from a traditional healer. TEM was generally free, 169 (90%) reported not to have spent any money to obtain it.

The qualitative study involved a total of 38 participants: 11 traditional healers, 21 MK patients who had used TEM and 6 MK patients who had not used TEM. The baseline characteristics of these individuals are presented in Table 4. Overall, it was a mix of male and female, young and old, not educated and highly educated. In addition, three informal group discussions (IGDs) were conducted, each with around 15 participants (these were naturally composed groups of patients who had used or not used TEM, relatives and friends).

**Table 4.** Baseline characteristics of people who participated in the in-depth interviews, including traditional healers and patients with microbial keratitis (both traditional eye medicine (TEM) users and non-users).

Participant	Age	Sex	Marital status	Occupation	Household size	Education	Religion
Traditional Healers (n=11)							
**1**	70	Male	Divorced	Farmer	1	None	Christian
**2**	56	Female	Married	Farmer	4	None	Christian
**3**	52	Female	Widowed	Farmer	3	None	Christian
**4**	76	Female	Married	Farmer	8	Primary	Christian
**5**	78	Female	Married	Farmer	5	-	-
**6**	53	Female	Widowed	Farmer	2	-	Christian
**7**	72	Female	Widowed	TBA	4	Primary	Christian
**8**	82	Male	Divorced	Farmer	8	None	Christian
**9**	59	Male	Married	Carpenter	18	Secondary	Christian
**10**	69	Female	Married	TBA	6	Primary	Christian
**11**	60	Female	Widowed	TBA	5	Primary	Christian
TEM Users (n=21)							
**1**	42	Male	Married	Farmer	7	Primary	Christian
**2**	46	Male	Married	Charcoal maker	8	Primary	Christian
**3**	26	Male	Married	Mechanic	4	Primary	Christian
**4**	53	Female	Married	Farmer	5	Primary	Christian
**5**	38	Female	Married	Farmer	3	Primary	Christian
**6**	26	Male	Single	Graduate	5	Tertiary	Christian
**7**	18	Female	Single	Farmer	6	Secondary	Christian
**8**	39	Male	Married	Farmer	5	None	Muslim
**9**	85	Female	Widowed	Farmer	18	None	Christian
**10**	60	Female	Married	Business	5	None	Christian
**11**	72	Female	Married	Farmer	8	None	Christian
**12**	29	Male	Married	Teacher	3	Tertiary	Christian
**13**	60	Male	Married	Farmer	6	Primary	Muslim
**14**	39	Female	Married	Farmer	5	Primary	Christian
**15**	54	Male	Married	Guard	4	Primary	Christian
**16**	58	Female	Married	Farmer	4	Primary	Christian
**17**	30	Female	Divorced	Farmer	4	Primary	Christian
**18**	81	Male	Married	Farmer	9	None	Christian
**19**	81	Male	Married	Farmer	5	Primary	Christian
**20**	69	Male	Married	Farmer	17	Primary	Christian
**21**	20	Male	Single	Shop keeper	20	Primary	Muslim
TEM Non-Users (n=6)							
**1**	56	Male	Married	Teacher	6	Tertiary	Christian
**2**	25	Male	Married	Bike rider	6	Primary	Christian
**3**	39	Male	Married	Accountant	1	Tertiary	Christian
**4**	30	Female	Single	Hairdresser	1	Primary	Christian
**5**	20	Male	Single	Farmer	10	Secondary	Christian
**6**	19	Female	Single	Student	4	Tertiary	Muslim

TBA: Traditional Birth Attendant;

The major factors coming out as the reasons for using TEM included lack of consumer confidence in conventional medicine, health system breakdown, poverty, fear, cultural belief in TEM, Role of Traditional Healers, personal circumstances and Ignorance.

### Lack of confidence in conventional medicine

While some participants reported visiting health centres for treatment, many talked of resorting to TEM with the persistence in pain after use of conventional medicine. A 26-year male mechanic said “ *At first, I got some relief when I put the eye drop, but later, it pained me severely and I was advised to use herbs. Having seen no great improvement, I started using herbs*.” A participant in an IGD told us “ *We are using western medicine to no avail. You can use western medicine for a week or a month but don’t get healed.”* A 75-year male traditional healer reported that “ *many people with eye problems come to me because some even fail to get cured from Mbarara hospital and are referred to me. I then put my traditional eye medicine like twice and they gain or enjoy life again.”* These statements supported the observation above that the majority (73%) of the TEM users had applied it after they had visited a health facility.

### Lack of service in health facilities

Inadequate care including lack of medicines, rude health workers, unskilled health workers and poorly equipped health facilities, especially government owned ones, were reported as major drivers to use of TEM by a majority of patients. *“There are no experts or doctors experienced in treating eye diseases in Health Centres within our vicinities. When you find a doctor at a Health Centre, they say that they don’t know such an eye disease you are suffering from”* (a 28-year unemployed man). The majority of primary health facilities do not have trained primary eye care workers. Eye patients are reviewed by general health workers who may have limited experience with managing ophthalmic condition. Eye care workers are nurses who have received an ophthalmic certificate course in examination and management of common eye conditions. In addition, as an 81-year-old farmer put it *“Health facilities within our areas don’t have eye medicine, examination machines and they are also unwelcoming to a person who has gone there. One just looks at the eye, prescribes the medicine and start treating the illness. Or, you hear medicine has been brought but when you go there the next day, you are told there is no medicine.”*

### Poverty as a barrier to access care

With subsistence farmers constituting the major part of the population, poverty was reported as a key barrier to accessing eye care, encouraging people to opt for TEM. This was expressed as being unable to afford transport to eye hospitals and treatment. In an IGD1, one respondent told us *“Those of us who are able to afford treatment are very few you can count them; many people who have the same problem have turned blind because they cannot afford treatment.”* Another person added *“It’s a result of poverty! Many people in the village have no money. Even sometimes you don’t have money in the pocket, so you pick the herb and apply it to the sick eye. You get to come here at the facility when you can’t count the types of herbs you have tried just because of poverty.”* Compared to going to hospital and the costs involved, TEM was a far cheaper option: the majority of the patients had obtained it from within their homesteads and had not spent any money on it.

### Fear of the eye hospital

Most people lived far from the eye hospital and fear of travelling long distances, which was reported as a constraint. *“One can be having money but chooses not come to the hospital fearing how he will reach. Not all people are poor, but one just wonders where he is to pass and continue to Mbarara eye hospital. There are reluctant for example one says he won’t be able to reach the place he has never gone to”* (an 81-year old male farmer from a distant village). We found that most of the patients travelled l distances (about 90 km) to reach the only referral eye hospitals in Mbarara town. Another form of fear was of what treatment would be offered; some people thought that this would make them go blind. For example, a participant in IGD2 told us *“What stops them from going to the hospital is that one is told they are going to operate your eye and after that it means that it is damaged completely you will never see again. That is the reason many people fear coming to the hospital, they say when you are operated the eye ends up getting damaged. They say when you reach in the hospital and get operated, it doesn’t get well”*

### Cultural understanding of MK and its treatment

Use of TEM in general is viewed as an acceptable practice and as part of culture in the community. It was revealed by several participants that MK is culturally understood as a disease to be treated locally. Almost all participants talked of receiving advice to use TEM from fellow community members who attest that it cured them. An 81-year old female farmer told us *“People in communities don’t know that MK as an eye disease is treated in hospitals or that there are hospitals that can treat it. People say it is cured by traditional eye medicine.”* Another 42-year old farmer said *“The old people we live with know those medicines and they testify that they cured them. Therefore, they encourage one who is suffering from an eye disease to keep using them saying he too will get well.*” Most of the people came from rural settings where there is a strong sense of community.

### Belief in TEM

From the experience of previous TEM users and personal experience of use, it was not surprising that almost all participants who had used TEM believed it was effective. They attributed their failure to heal to their body makeup. “ *The old people believe and know that traditional eye medicine cures eye diseases. There are people, they identified for me who used the same medicine and got well. Even themselves, they told me that they used it and got cured”* (a 42-year male farmer). “ *The person who gave me traditional eye medicine told me she too suffered from the same disease and got healed by the same herbs*” (a 60-year old butter maker). On being asked why it had not worked for them, a 53-year old female farmer responded “ *those who don’t heal I think the condition of the eye might have needed medical attention from doctors as genetically people are different. There is one who heals by traditional eye medicine and another who doesn’t and is only treated by modern medicine from hospitals.”*

### Role of traditional healers

With the belief and acceptance that use of TEM is within their culture, many had confidence in traditional healers. The traditional healers themselves also had a strong confidence in their medicine and reported remarkable cure rates. One 56-year old traditional healer said: “ *They go to the hospitals and come back to me when they have failed to heal with modern medicine. I give them traditional eye medicine and they get healed, none that I have treated or given my medicine has failed to get well*” Another 75-year old male healer reported “ *There are many people I have treated; none I gave my medicine has ever complained that it failed to heal her or him. Whoever I meet just praises God and prays for me to be blessed. I treat people with faith in God.”*

### Personal circumstances

Desperation due to the pain of the condition and the view of TEM as a form of first aid was mentioned as a prompt to use traditional medicine. This was mostly reported among patients who used TEM before presenting to health facilities. Participants explained that with the pain, one can use anything recommended to him or her to the extent of accepting TEM containing needle prick blood from another person without being afraid of contracting HIV. A 42-year male farmer told us *“This disease is so painful. No one should suffer from it because, with pain you can use anything given to you. You are not mindful of HIV, you only want the pain gone”.* A 85-year female farmer wondered, “ *Can anyone who has been found in pain and recommended an herb fail to use it? Pain can make you do anything*”.

### Lack of awareness to the dangers of TEM

Interestingly, most participants did not think using TEM could be dangerous. “ *Traditional eye medicine doesn’t damage the eye, it just rinses or cleanses it*” (a 46-year old male charcoal burner). “ *There are no risks of using traditional eye medicine because when one fails to get healed, she or he goes somewhere else or to hospitals*” (an 85-year female farmer). In addition, some thought it was better than conventional medicine and did not have any side effects like most conventional medicines. A 59-year old traditional healer said, “ *Our herbal medicine is fresh not preserved.”*

## Discussion

This study investigated the extent of TEM use by people with microbial keratitis, and how this impacts their clinical presentation and outcome. We went on to explore more deeply the specific practices and the reasons and beliefs behind using TEM. The use of TEM in Southern Uganda in the treatment of MK is common (60%), and more frequent than that previously reported from Malawi (34%) and Tanzania (25%) ^9, 10^. Importantly, we found that people who used TEM presented later with a more severe clinical picture and they ended up with worse final visual acuity outcomes at 3-months, compared to those who had not used TEM.

Our findings are similar to previous reports from Malawi, which found that patients who had used TEM presented later than those who had not used TEM ^9, 16^. The previous studies, however, did not examine final outcomes, after the infection had been treated. MK is a disease where prompt treatment is critical if one is to improve the likelihood of a good outcome. We know from prior literature that once an infection is advanced, treatment does relatively little to change its course ^17^. The clear conclusion from earlier studies from South Asia and East Africa is that effective treatment of MK should be started as early as possible to save the eye and achieve the best possible outcomes ^18, 19^.

In this study we combined both quantitative modelling approaches and complementary qualitative approaches to investigate not only “what” but also “why” people use TEM. In the explanatory multivariable model, increasing distance to the eye hospital, lower education level, an onset not linked to trauma and not being married were associated with TEM use. These were explored further in the informal group discussions (IGDs). These discussions the major reported reasons for using TEM were around consumer confidence in the health system, access, poverty and cultural influence.

Importantly, we found that most people who used TEM did so after first visiting a government health facility. This is consistent with the IGDs, in which people felt that conventional medicine was not helping, leading them to resort to alternative approaches. This conclusion could be a result of inappropriate treatment. However, even with appropriate treatment, the clinical response can be slow, especially for fungal keratitis. Patients need to be properly counselled to manage expectations. Another important aspect is good pain management on top of the anti-microbial treatment. Patients reported that desperation due to pain made them more likely to try many options to find relief. This initial early contact point with the formal health system represents an opportunity to improve the diagnosis and treatment of people with MK, through providing enhanced training, diagnostic tools and medication in the primary care setting.

Lack of appropriate ophthalmic medicines is a major challenge. For example, the best current evidence indicates that topical natamycin is the treatment of choice for filamentous fungal keratitis ^20^. However, this is currently not readily available in the main ophthalmic units Uganda or elsewhere in SSA. It is certainly not available in more isolated locations. Therefore, patients with a fungal MK will not access effective treatment until they arrive in a major eye unit. Natamycin was added to the WHO Essential Medicines List in 2018, which will hopefully result in greater availability soon.

Limited access to eye care was a major driver of TEM use. This was evident in the regression modelling, with increasing TEM use with increasing distance to the eye hospitals. The majority of TEM users came from districts relatively far away where no eye care facilities were situated. This was a strong and frequently articulated theme in the interviews and discussions. Multiple people commented on the lack of eye health services in the nearby health facilities, the long distances to the eye hospital and poverty is a major barrier to access (because of the high transport and other direct costs). Several people also highlighted that government health centres near to them have no eye specialists or treatment and do not treat eye conditions. Pharmacies simply sell available eye drop medication, with no examination; frequently these are steroid and antibiotic combinations which may result in more harm than good in fungal keratitis. Unfortunately, Uganda still grapples with a severe shortage of human resources and infrastructure for eye health ^21^.

Although the regression model did not demonstrate a relationship between economic status and TEM Use, during the IGDs poverty was reported to be a major driver for using TEM. In the model, there were only a handful of people in the upper economic status which may have obscured this relationship. The majority of the patients were subsistence farmers and therefore not able to readily afford the cost of medicines and transportation. In contrast, TEM could be accessed closer to home at almost no cost. Most of the patients used got the TEM from their nearby gardens or from the neighbour and applied it freshly squeezed into the eye. People who are married may have access to greater household financial resources, possibly explaining why being married was associated with less TEM use.

We found that TEM use was linked to strong cultural beliefs and this seemed related to the level of education. In the model, people with no or little education were more likely to use TEM. It was worrying that people did not perceive TEM use as potentially dangerous. This was also reinforced by messages from traditional healers and older members of the community who carry a high level of respect. Public health orientated messaging and health education need to particularly focus on and work with these groups. There is some evidence from Malawi and Nigeria, where ophthalmologists worked with traditional healers to lower the use of TEM, that changes are possible ^7, 16^. Although, in our context, only 3% of TEM users consulted a traditional healer, their place in society cannot be underestimated and it would be in our best interest to bring them on board.

### Strengths/limitations

The use of a mixed methods approach provided a more informative data on reasons for using TEM for MK in Uganda. To the best of our knowledge, this was the first study in SSA that looked at 3-month outcomes of people who had used TEM for treatment of MK. Although a sensitive topic, it was noted that participants and traditional healers were willing to talk about their TEM experiences. We did not have any evidence that people withheld information. The large numbers were enough to have a well powered study to explore factors associated with TEM use. Inclusion of children would have provided a more overall understanding of this topic, however, this was not practical in out setting.

## Conclusion

TEM use is an important factor in the presentation and outcome of MK in Uganda, leading to delayed presentation to hospital, a poor presentation and a worse outcome. Cultural beliefs, access to the health system (due to poverty and long distances) and inherent challenges in the primary health centres (lack of knowledge, medicines, equipment and supplies) are major drivers of TEM use. Sensitisation of the people and capacity building in the primary health centres will be a step in the right direction to mitigate these effects.

## Data availability

### Underlying data

Havard dataverse: Traditional Eye Medicine use in Microbial Keratitis in Uganda. https://doi.org/10.7910/DVN/5GOPKZ ^14^.

This project contains the following underlying data:

- tem_data_descriptive_5May2019.tab (quantitative underlying data)
- tem_coding_framework_May2019.tab (codes of qualitative data responses)

### Extended data

Havard dataverse: “Topic guides for exploring Traditional Eye Medicine Use for treatment of Microbial Keratitis in Uganda.docx”, Traditional Eye Medicine use in Microbial Keratitis in Uganda, https://doi.org/10.7910/DVN/5GOPKZ ^14^.

This project contains the following underlying data

- Topic guides for exploring Traditional Eye Medicine Use for treatment of Microbial Keratitis in Uganda.docx (Topic guides that were used to probe respondents to talk about their understanding, opinions and experiences of using Traditional Eye Medicine)
- Quantitative questionnaire on use of Traditional Eye Medicine.docx (A of a quantitative questionnaire that was used to collect information from all the patients with MK on their history of use of Traditional Eye Medicine)

Data are available under the terms of the Creative Commons Zero “No rights reserved” data waiver (CC0 1.0 Public domain dedication).
