# Supplementary material for: Traditional eye medicine use in microbial keratitis in Uganda: a mixed methods study
Source: Wellcome Open Res. 2019 Oct 4;4:89. Originally published 2019 Jun 6. [Version 2] doi: 10.12688/wellcomeopenres.15259.2 (PMC6784788; doi:10.12688/wellcomeopenres.15259.2)
Supplement: Peer review history

# Peer review: Reviewer response for version 2

**DOI:** 10.21956/wellcomeopenres.16901.r36670

**Published:** 2019-10-08

**Authors:** Savitri Sharma

**Affiliations:** 1 Jhaveri Microbiology Center, Brien Holden Eye Research Centre (BHERC), L V Prasad Eye Institute (LVPEI), Hyderabad, Telangana, India

Revision satisfactory.

I confirm that I have read this submission and believe that I have an appropriate level of expertise to confirm that it is of an acceptable scientific standard.

# Peer review: Reviewer response for version 1

**DOI:** 10.21956/wellcomeopenres.16656.r35735

**Published:** 2019-07-24

**Authors:** David Yorston

**Affiliations:** 1 Tennent Institute of Ophthalmology, Gartnavel Hospital, NHS Greater Glasgow and Clyde, Glasgow, UK

This is a useful addition to the mounting evidence that improving the early treatment of microbial keratitis should be a priority for prevention of blindness programmes.

- The authors conclude that TEM is more likely to be used if patients have less access to effective eye care facilities. Although poverty was cited by many participants as a driver for TEM use, it was not significant in the multivariate analysis. This may be explained by the paucity of higher SES patients in both groups. I think it is likely that poverty does contribute to TEM use, alongside the other factors.
- Although the text of the results section states that TEM users had worse presenting acuity than non TEM users, the data in Table 2 appears to contradict this, and I suspect there may be an error in the table.
- This study confirms the finding of previous authors who noted that TEM use is associated with a greater risk of hypopyon. The underlying assumption of this article is that all patients had microbial keratitis prior to TEM use. However, it is possible that some may have had self-limiting, or minor conditions, such as a corneal abrasion or conjunctivitis. The introduction of unsterile preparations on to a compromised ocular surface may have led to *de novo* development of microbial keratitis.
- An unexpected finding is that TEM use in this population was usually independent of traditional healers. I have always assumed that TEM use is partly driven by a desire for answers that western medicine is not good at providing, particularly "Why has this happened to me?". This study would seem to indicate that the main motivation for most patients was a simple desire for faster and greater improvement in their symptoms.
- A less surprising finding is that outcomes were significantly worse for patients using TEM. Previous studies have not been able to obtain outcome data, as it can be difficult for these patients to return for review. It is valuable to have clear evidence that TEM use is harmful.
- One significant weakness in the study is the exclusion of children. In Tanzania we found that 50% of TEM users were aged 11 or younger. I suspect that the findings would be similar in children and adults, but the authors should acknowledge this weakness in the discussion.
- The ready availability of TEM in people's homes and gardens means that campaigns to reduce the use of TEM are unlikely to be successful. Prevention of blindness programmes would be better to focus on improving the delivery of eyecare, and raising the quality of the care delivered. Anecdotally, I can report that TEM use was widespread in a poor part of rural Tanzania, but almost non-existent in the relatively developed Central Province of Kenya. My experience would appear to support the authors' conclusion that improving rural eye care will lead to a decline in the harms caused by TEM.

I confirm that I have read this submission and believe that I have an appropriate level of expertise to confirm that it is of an acceptable scientific standard.

# Response

**Published:** 2019-09-02

**Authors:** SIMON ARUNGA

**Comment:** The authors conclude that TEM is more likely to be used if patients have less access to effective eye care facilities. Although poverty was cited by many participants as a driver for TEM use, it was not significant in the multivariate analysis. This may be explained by the paucity of higher SES patients in both groups. I think it is likely that poverty does contribute to TEM use, alongside the other factors.

**Response: We agree with the reviewer that poverty does contribute to TEM use and was indeed reported by many participants. In the multivariable model, there were only a handful of people in the upper economic status which may have obscured this relationship. We have added this comment in lines 339-341. Also to note is that SES/Access/poverty are all on a similar/same causal path and do not function independently of each other.**

**Comment: **Although the text of the results section states that TEM users had worse presenting acuity than non TEM users, the data in Table 2 appears to contradict this, and I suspect there may be an error in the table.

**Response: We thank the reviewer for spotting this. We noticed that the data had been accidentally interchanged. It has been corrected in table 2.**

**Comment**: This study confirms the finding of previous authors who noted that TEM use is associated with a greater risk of hypopyon. The underlying assumption of this article is that all patients had microbial keratitis prior to TEM use. However, it is possible that some may have had self-limiting, or minor conditions, such as a corneal abrasion or conjunctivitis. The introduction of unsterile preparations on to a compromised ocular surface may have led to de novo development of microbial keratitis.

**Response: We agree with the reviewer and feel the same way. However, there was no way of objectively ascertaining this fact. We intend to explore this in our future studies.**

**Comment**: An unexpected finding is that TEM use in this population was usually independent of traditional healers. I have always assumed that TEM use is partly driven by a desire for answers that western medicine is not good at providing, particularly "Why has this happened to me?". This study would seem to indicate that the main motivation for most patients was a simple desire for faster and greater improvement in their symptoms.

**Response: Indeed, this was surprising. Only 3% of the participants visited a traditional healer to obtain TEM. From our further exploration of this in the qualitative studies, our impression is that *“everyone in the community is a traditional healer”* since the knowledge of the herbs is common among the community members. However, this does not negate the role of the healers since they are strong advocates for TEM use.**

**Comment**: A less surprising finding is that outcomes were significantly worse for patients using TEM. Previous studies have not been able to obtain outcome data, as it can be difficult for these patients to return for review. It is valuable to have clear evidence that TEM use is harmful.

**Response: We thank the reviewer for acknowledging this new contribution.**

**Comment**: One significant weakness in the study is the exclusion of children. In Tanzania we found that 50% of TEM users were aged 11 or younger. I suspect that the findings would be similar in children and adults, but the authors should acknowledge this weakness in the discussion.

**Response: We thank the author for this comment. Although we provided care for children who presented with Microbial Keratitis, the design of our study enrolled only adults due to pragmatic reasons such as being able to test people for HIV, subjecting children under general anaesthesia for corneal scrapping and ethical approvals for a vulnerable group. In addition, we found out during the pilot phase that microbial keratitis was not very common among children in our setting, accounting for only about 3% of all microbial keratitis cases. However, this point has been acknowledged in the limitation. lines 364-365.**

**Comment**: The ready availability of TEM in people's homes and gardens means that campaigns to reduce the use of TEM are unlikely to be successful. Prevention of blindness programmes would be better to focus on improving the delivery of eyecare, and raising the quality of the care delivered. Anecdotally, I can report that TEM use was widespread in a poor part of rural Tanzania, but almost non-existent in the relatively developed Central Province of Kenya. My experience would appear to support the authors' conclusion that improving rural eye care will lead to a decline in the harms caused by TEM.

**Response: We thank the reviewer for this comment.**

# Peer review: Reviewer response for version 1

**DOI:** 10.21956/wellcomeopenres.16656.r35736

**Published:** 2019-07-01

**Authors:** Savitri Sharma

**Affiliations:** 1 Jhaveri Microbiology Center, Brien Holden Eye Research Centre (BHERC), L V Prasad Eye Institute (LVPEI), Hyderabad, Telangana, India

This report gives an account of the "mystery" surrounding the traditional eye medicine usage in the treatment of microbial keratitis. The practice is rampant in some of the developing and underdeveloped countries and the more we know and understand this practice better we can get at influencing people to make a distance from them. Social, cultural, economical and emotional factors - all seem to be responsible for continued presence of this unwanted practice. This reviewer appreciates the efforts of the authors in putting up this paper together which is very well written. Following are minor comments that may help make the paper even better:

1. Abstract: Results begins with digits which in good writing should be avoided and replaced with words.
2. Methods: Clinical examination and microbiological methods are not described at all. A description would allow better understanding of how the data was collected.
3. Analysis, Page 4, results, last but one line: The word farmer is spelt wrongly with one "r" missing.
4. There is no data on what type of organisms were involved in the microbial keratitis in the two study groups. If microbiology was done, as is claimed in methods, there should be results of the same. Similarly, how were the patients treated in the control group that did not receive traditional eye medicine? These are important determinants of the outcome in the two groups that have been compared. My comments of "partly satisfied" are related to these issues.
5. Discussion: Para 2, line 6: “...if one is improve the likelihood of a good outcome.” This sentence is incorrect with a missing word "to".

I confirm that I have read this submission and believe that I have an appropriate level of expertise to confirm that it is of an acceptable scientific standard, however I have significant reservations, as outlined above.

# Response

**Published:** 2019-09-02

**Authors:** SIMON ARUNGA

**Comment**: Abstract: Results begins with digits which in good writing should be avoided and replaced with words.

**Response: We thank the reviewer for spotting this. We have revised this sentence to read “Out of 313 participants enrolled, 188 reported TEM use”.**

**Comment:** Methods: Clinical examination and microbiological methods are not described at all. A description would allow better understanding of how the data was collected.

**Response: We thank the reviewer for this comment. The detailed assessment of the patients has been described in a different report (under review), however, we have revised the manuscript and summarised patient assessment. Line 48-55.**

**Comment:** Analysis, Page 4, results, last but one line: The word farmer is spelt wrongly with one "r" missing.

**Response: We thank the author for spotting this. It has been corrected. Line 115.**

**Comment:** There is no data on what type of organisms were involved in the microbial keratitis in the two study groups. If microbiology was done, as is claimed in methods, there should be results of the same. Similarly, how were the patients treated in the control group that did not receive traditional eye medicine? These are important determinants of the outcome in the two groups that have been compared. My comments of "partly satisfied" are related to these issues.

**Response: We would like to draw the attention of the reviewer to the last section of table 2 which summarises the types of organisms in the two groups. Although the proportion of fungal keratitis was more common among the people who had used TEM, the evidence of this difference was weak. We agree with the reviewer that treatment for people with keratitis should consider the history of use of TEM since that could influence the organisms involved, especially in the absence of a good microbiology support. However, treatment of the participants in our study was dependant on the microbiological findings.**

**Comment:** Discussion: Para 2, line 6: “...if one is improve the likelihood of a good outcome.” This sentence is incorrect with a missing word "to"

**Response: We thank the author for spotting this. It has been corrected. Line 296.**
